# Prediction of Body Fluids where Proteins are Secreted into Based on Protein Interaction Network

**DOI:** 10.1371/journal.pone.0022989

**Published:** 2011-07-29

**Authors:** Le-Le Hu, Tao Huang, Yu-Dong Cai, Kuo-Chen Chou

**Affiliations:** 1 Institute of Systems Biology, Shanghai University, Shanghai, China; 2 Department of Chemistry, College of Sciences, Shanghai University, Shanghai, China; 3 Key Laboratory of Systems Biology, Shanghai Institutes for Biological Sciences, Chinese Academy of Sciences, Shanghai, China; 4 Shanghai Center for Bioinformation Technology, Shanghai, China; 5 Centre for Computational Systems Biology, Fudan University, Shanghai, China; 6 Gordon Life Science Institute, San Diego, California, United States of America; Semmelweis University, Hungary

## Abstract

Determining the body fluids where secreted proteins can be secreted into is important for protein function annotation and disease biomarker discovery. In this study, we developed a network-based method to predict which kind of body fluids human proteins can be secreted into. For a newly constructed benchmark dataset that consists of 529 human-secreted proteins, the prediction accuracy for the most possible body fluid location predicted by our method via the jackknife test was 79.02%, significantly higher than the success rate by a random guess (29.36%). The likelihood that the predicted body fluids of the first four orders contain all the true body fluids where the proteins can be secreted into is 62.94%. Our method was further demonstrated with two independent datasets: one contains 57 proteins that can be secreted into blood; while the other contains 61 proteins that can be secreted into plasma/serum and were possible biomarkers associated with various cancers. For the 57 proteins in first dataset, 55 were correctly predicted as blood-secrete proteins. For the 61 proteins in the second dataset, 58 were predicted to be most possible in plasma/serum. These encouraging results indicate that the network-based prediction method is quite promising. It is anticipated that the method will benefit the relevant areas for both basic research and drug development.

## Introduction

Protein secretion is a universal biological process occurring in all organisms. Secreted proteins such as hormones, digestive enzymes, neurotransmitters as well as antibodies, play vital regulatory roles in various biological activities such as reproduction, digestion, nerve conduction and immunization [1]. The studies on the secreted proteins under different physiological and pathological conditions in different growth and development stages can deepen the understanding of many biological phenomena. Under the condition of the disease, some secreted proteins showed abnormal concentration level [2]. In recent years, several genes encoding secreted proteins have been found to be consistently over-expressed in various cancer specimens [3], [4], [5]. For example, MIC1 gene has been observed to be over-expressed in breast, colorectal and prostate cancer patients [5]. These proteins that could be detected in blood, urine or other body fluids are more suitable to serve as biomarkers for diagnosis [6]. This is because the body fluid test (e.g. blood test or urine test) is less invasive, cheaper, and easier to collect and process samples than tissue biopsy test [7], [8] since the latter requires surgery to get the disease tissues. Besides, identification of body fluids where proteins can be secreted into is very helpful for protein function annotation and biomarker discovery.

However, how to realize the identification is still a big challenge even having the advanced proteomics technologies because there are a large amount of proteins with a variety of modifications in body fluids [8]. To address this problem, let us resort to computational approaches. In the past two decades, many studies have focused on predicting the subcellular locations of proteins in both prokaryotes and eukaryotes (see, e.g., [9], [10], [11], [12], [13], [14], [15], [16], [17], [18], [19], [20], [21] as well as a long list of the relevant references in a comprehensive review [22]). Unfortunately, none of the aforementioned methods was aimed at identifying the final locations where the extracellular proteins are secreted. The present study was initiated in an attempt to address this problem, with a focus on human secreted proteins and a novel approach via protein-protein interaction (PPI) network.

According to a recent comprehensive review [23], to establish a really useful statistical predictor for a protein system, we need to consider the following procedures: (i) construct or select a valid benchmark dataset to train and test the predictor; (ii) formulate the protein samples with an effective mathematical expression that can truly reflect their intrinsic correlation with the attribute to be predicted; (iii) introduce or develop a powerful algorithm (or engine) to operate the prediction; (iv) properly perform cross-validation tests to objectively evaluate the anticipated accuracy of the predictor. Below, let us describe how to deal with these steps.

## Materials and Methods

### Training dataset

The human secreted proteins were retrieved from UniProt [24]. The detailed procedures for collecting the human secreted protein sequences are as follows. **(1)** Open the web-page at http://www.uniprot.org/ (Release 2011_05). **(2)** Click the button “Fields”, followed by selecting “Subcellular location” for 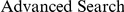, “Secreted” for 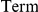, “Homo sapiens” for 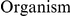, and “Experimental” for 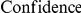. **(3)** Click Add & Search. Thus we collected a total of 1,019 experiment-validated human secreted proteins. Subsequently, these proteins were mapped to 11 different kinds of body fluids contained in the human body fluid database “Sys-BodyFluid” [25] (http://lifecenter.sgst.cn/bodyfluid/), where the body fluid proteome data was manually collected from 50 peer-review publications. Finally, a total of 682 human proteins have been obtained that can be secreted into the aforementioned body fluids.

The human protein-protein interaction (PPI) networks were retrieved from STRING [26], [27] (http://string.embl.de/), which is a database dedicated to both physical and functional interactions. Information derived from 3 kinds of sources (high-throughput experiments, mining of databases and literature, and prediction from genomic context analysis) was integrated into several PPI networks. As done by previous investigators in using the intuitive graphic representation to deal with complicated biological systems, such as enzyme-catalyzed system [28], [29], [30], protein-folding system [31], and drug metabolism system [32], here the PPI network can also be intuitively expressed via a graph, in which each of the proteins is represented by a node, and the interaction is represented by the edge between two nodes. The edge is weighted by the interaction confidence, i.e., the likelihood that the interaction exists between two nodes. The interaction confidence score of two proteins is obtained as follows: first, the interactions from each source were scored by benchmarking them against a common reference set; then these scores were combined in the naive Bayesian fashion [26].

Of the 682 human secreted proteins, we have found that 153 proteins have no PPI information nor interact with any of the other secreted proteins, while 529 proteins interact with at least one of the other proteins in the human PPI network from STRING. Thus, we obtained a working PPI network that consists of 529 nodes (proteins) and 27,176 interaction units. Such 529 human secreted proteins in the newly constructed PPI network were used as the training dataset for developing the current network-based method.

**Figure 1:**
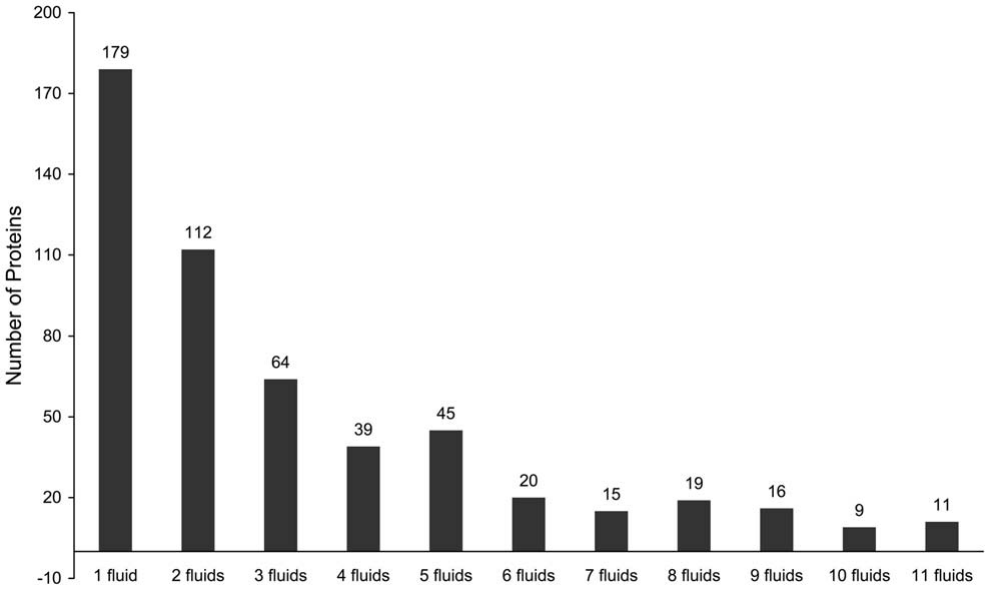
The numbers of proteins that are secreted in different types of body fluids. See Table 1 for the definition of the numerical codes used here for the body fluid types.

The distribution of the 529 human secreted proteins classified according to the 11 different types of body fluids is shown in **Table 1**, from which we can see that the sum of numbers in column 3 is 1708 that is much more than 529, the number of secreted proteins. This is because many proteins can be secreted into more than one body fluid [25], as illustrated in **Figure 1**. As we can see from the figure, of the 529 human secreted proteins, 179 can be secreted into one body fluid, and 350 proteins can be secreted into two or more different types of body fluids. Therefore, we are to deal with a multi-label classification problem.

**Table 1:** A breakdown of the 529 human secreted proteins in the training dataset according to the 11 different types of body fluids into which they can be secreted.

Type	Body fluid	Number of proteins in dataset
1	Amniotic fluid	192
2	Bronchoalveolar lavage fluid	65
3	Cerebrospinal fluid	204
4	Milk	71
5	Nipple aspiration fluid	37
6	Plasma/Serum	418
7	Saliva	175
8	Seminal fluid	155
9	Synovial fluid	63
10	Tear	84
11	Urine	244
Sum		1,708

### Testing datasets

Two testing datasets were used in this study. The first one contains 57 blood-secreted proteins, which was obtained as follows. First of all, 305 blood-secreted proteins were retrieved from the positive dataset in [33], where the proteins met the criteria that they were not only secreted but also serum/plasma detected. Of the 305 proteins thus obtained, 172 were excluded because they occurred the training dataset, and 76 proteins were also excluded because they had no interaction with the proteins in the training dataset and hence could not be processed by the current method (see the Network-based Method section). The remaining 57 blood-secreted proteins were used to test our method (**Table S1**).

The second testing dataset contains 61 proteins as obtained as follows. From [33], we first collected 122 abnormally expressed proteins involved with various cancers as indicated by many published proteomics studies. From these proteins, we obtained 77 plasma/serum secreted proteins. After removing those that had been contained in the training dataset and those that had no interaction with the proteins in the training dataset, we finally obtained the remaining 61 possible marker proteins (**Table S2**) for the second testing dataset.

### Network-based method

Many interacting proteins must co-occur in the same location to participate in the biological processes [34]. Accordingly, we can presume that the interacting secreted proteins are likely to be secreted into the same body fluids. In other words, the following assumptions would be valid.

Given a query protein, the higher interaction confidence score between it and its interacting counterpart, the more likely they are to be secreted into the same body fluid. Also, the more its interacting proteins in a certain body fluid, the more likely it is to be secreted into such body fluid [35]. With these points in mind, the body fluids that secreted proteins can be secreted into can be predicted as follows.

First, let us denote the *n* proteins in the PPI network as 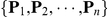 and the 11 body fluids as 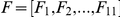, where 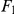 stands for the “Amniotic fluid”, 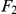 the “Bronchoalveolar lavage fluid”, 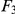 the “Cerebrospinal fluid”, and so forth (cf. **Table 1**). Thus, the body fluids that the proteins in the PPI network is secreted into can be described as
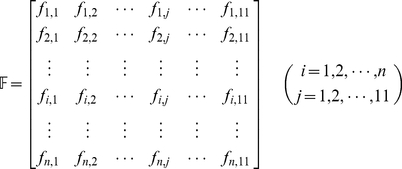
(1)where
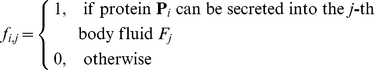
(2)For several query proteins 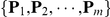, their interactions with the *n* proteins in the PPI network can be described as
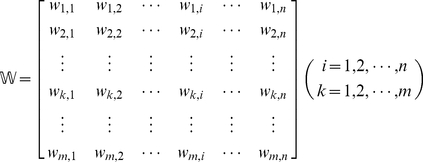
(3)where 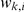 denotes the interaction confidence score [27] between 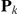 and 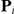. If there is no interaction between 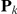 and 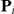, we have 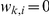. Since no self-interaction exists in the PPI network, 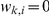 if 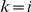. Now, let us use 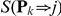
to denote the likelihood that the query protein 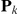 is secreted into the *j*-th body fluid 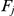. Thus, the likelihood that the 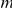 query proteins are secreted into the 11 body fluids can be formulated as
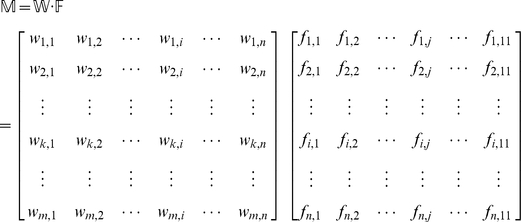


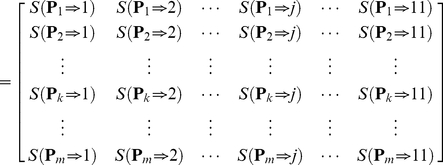
(4)where
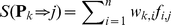
(5)The 11 elements of each row in Eq.4 represent the likelihoods that protein 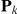 is secreted into the 11 body fluids, respectively. It can be seen from Eq.5 that the likelihood 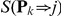 can be formulated as the sum of the interaction confidence scores of the protein 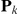 with its interacting proteins that can be secreted into the *j*-th body fluid 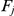. Such scoring approach takes both the interaction confidence score and the number of the interacting proteins into consideration, just like the weighted vote. Obviously, the higher the score, the more likely 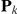 is to be secreted into the *j*-th body fluid 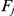. In Eq.4, the 11 scores in the 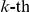 row for the query protein 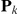 are used to reflect the likelihoods that it is secreted into the 11 body fluids, respectively. Accordingly, the most likely body fluid 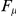 where 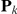 is secreted should be the one with the maximum score, as can be formulated below
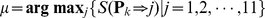
(6)where 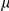 is the 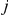 that maximizes the value of 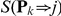.

Since many secreted proteins can be secreted into more than one body fluid, our method is dedicated to provide flexible information by predicting possible body fluids for secreted proteins, rather than the most likely body fluid. To realize this, let us sort the 11 elements of each row in Eq.4 according to descending order. By doing so, we obtain a 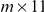 matrix as formulated by
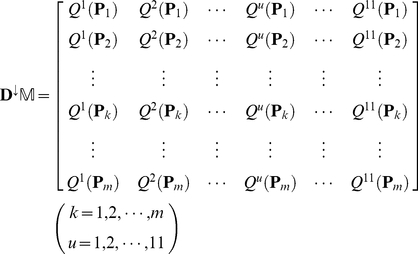
(7)where 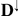 is a descending operator that arranges the 11 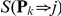 of each row in Eq.4 in descending order: 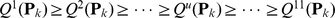. If two or more elements of the row in Eq.4 are equal to one another, they will be sorted in random order. Accordingly, the predicted results for the secreted protein 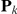 can be obtained from the descending order. For instance, if 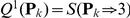, 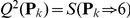, and 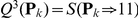, then that the query protein 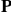 is secreted into the 3^rd^ body fluid (Cerebrospinal fluid) will have the maximum likelihood (cf. **Table 1**), that 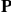 is secreted into the 6^th^ body fluid (Plasma/Serum) will have the second maximum likelihood, and that 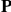 is secreted into the 11^th^ body fluid (Urine) will have the third maximum likelihood. And so forth. The predicted results thus obtained are called the 1^st^ order predicted body fluid, the 2^nd^ order predicted body fluid, the 3^rd^ order predicted body fluid, and so forth.

### Validation and Demonstration

In statistical prediction, the following three cross-validation methods are often used to examine a predictor for its effectiveness in practical application: independent dataset test, subsampling test, and jackknife test [36]. However, of the three test methods, the jackknife test is deemed the most objective [37], [38]. The reasons are as follows. **(1)** For the independent dataset test, although all the proteins used to test the predictor are outside the training dataset used to train it so as to exclude the “memory” effect or bias, the way of how to select the independent proteins to test the predictor could be quite arbitrary unless the number of independent proteins is sufficiently large. This kind of arbitrariness might result in completely different conclusions. For instance, a predictor achieving a higher success rate than the other predictor for a given independent testing dataset might fail to keep so when tested by another independent testing dataset [36]. **(2)** For the subsampling test, the concrete procedure usually used in literatures is the 5-fold, 7-fold or 10-fold cross-validation. The problem with this kind of subsampling test is that the number of possible selections in dividing a benchmark dataset is an astronomical figure even for a very simple dataset, as demonstrated by Eqs.28–30 in [23]. Therefore, in any actual subsampling cross-validation tests, only an extremely small fraction of the possible selections are taken into account. Since different selections will always lead to different results even for a same benchmark dataset and a same predictor, the subsampling test cannot avoid the arbitrariness either. A test method unable to yield a unique outcome cannot be deemed as a good one. **(3)** In the jackknife test, all the proteins in the benchmark dataset will be singled out one-by-one and tested by the predictor trained by the remaining protein samples. During the process of jackknifing, both the training dataset and testing dataset are actually open, and each protein sample will be in turn moved between the two. The jackknife test can exclude the “memory” effect. Also, the arbitrariness problem as mentioned above for the independent dataset test and subsampling test can be avoided because the outcome obtained by the jackknife cross-validation is always unique for a given benchmark dataset. Accordingly, the jackknife test has been increasingly and widely used by those investigators with strong math background to examine the quality of various predictors (see, e.g., [39], [40], [41], [42], [43], [44], [45], [46], [47], [48]). In view of this, here the jackknife cross-validation was also used to examine the prediction quality of the network-based method. Meanwhile, just for a demonstration to show biologists how to use the predictor for practical application, we also performed the computation for some independent datasets.

For the *j*-th order prediction, the accuracy 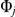 obtained by the jackknife test can be formulated as
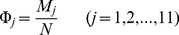
(8)where 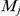 represents the number of the secreted proteins whose *j*-th order predicted body fluid is one of the true body fluids where the protein is secreted, and 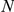 represents the total number of proteins in the PPI network. These 11-order jackknife cross-validation accuracies were used as an evaluation for the network-based method. According to Eq.8, high 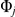 with small 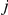 and low 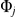 with large 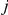 will indicate a good prediction based on the current prediction method.

In the PPI network, the average number of body fluids that each secreted protein is secreted into can be calculated by
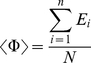
(9)where 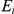 represents the number of body fluids that the secreted protein 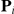 is secreted into. Hence, a new evaluation for the network-based method was proposed to calculate the likelihood that the first *k* order predicted body fluids contain all the true body fluids that the proteins can be secreted; it can be formulated as
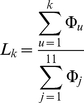
(10)where 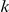 represents the smallest integer equal or greater than 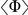 of Eq.9. Also, a large 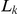 indicates a good prediction of the network-based method.

## Results and Discussion

### Performance of network-based method

**Figure 2:**
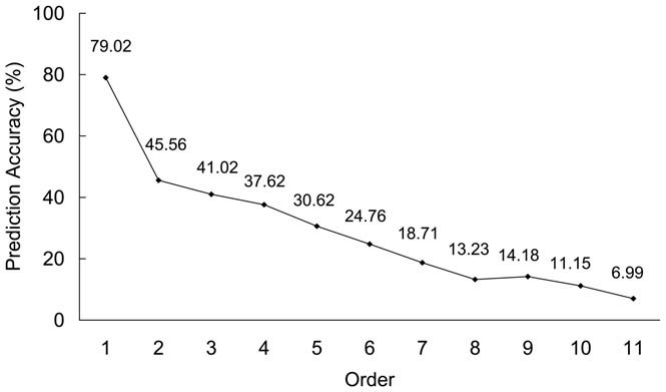
All the 11 order jackknife cross-validation accuracies by the network-based method for the 529 human secreted proteins.

In this study, the network-based method was applied to the 529 human secreted proteins to predict the body fluids where they were secreted. All the 11 order jackknife cross-validation accuracies are shown in **Figure 2**. From the downward-slope curve, we can see that except the 8^th^-order prediction accuracy, all the other higher-order prediction accuracies are higher than the lower-order ones, indicting that the body fluids were well prioritized for the proteins by the method. The 1^st^-order (most likely) prediction accuracy is 79.02%, indicating that the 1^st^-order predicted body fluid for the secreted proteins is believable. The 11^th^-order (least likely) prediction accuracy is 6.99%, indicting that the likelihood that the query protein is secreted into the 11^th^-order predicted body fluid is very low and such predicted body fluid can be ignored.

The average number of body fluids that each secreted protein in the PPI network can be secreted into is 3.23 according to Eq.9. Hence, a random guess of body fluid for the secreted proteins will have a 29.36% (3.23/11) success rate, lower than the first 5 order prediction accuracies. The parameter *k* (cf. Eq.10) was set to be 4 ([3.23]+1), i.e., we consider the first 4 order predicted body fluids from the 11 order prediction. The likelihood that the first 4 order predicted body fluids contain all the true body fluids that the proteins can be secreted into is 0.6294 according to Eq.10, indicating that the first 4 order predicted body fluids should be paid more attention to than others in the 11 predicted body fluids.

### The availability of using the PPI information to predict the body fluids that secreted proteins can be secreted into

Many important biological activities are mediated by proteins interactions. The interacting proteins should co-occur spatially and temporally to intact with each other [34]. Similarly, the interacting secreted proteins often are secreted into the same body fluids to perform their functions. For example, peptidoglycan recognition protein 1 (O75594, UniProt Protein) can be secreted into plasma/serum [49], saliva [50], [51], and urine [52], [53], [54]. Its interactions with the other proteins are shown in **Table 2**. Except 3 proteins (P07492, Q13410, and P05814), the other 20 neighbor proteins can be secreted into the plasma/serum or saliva or urine just like peptidoglycan recognition protein 1. According to the prediction criteria, when peptidoglycan recognition protein 1 was considered as a query protein, the first three order predicted body fluids that peptidoglycan recognition protein 1 can be secreted into are plasma/serum, saliva, and urine, which are consistent with the real locations.

**Table 2:** Interactions of peptidoglycan recognition protein 1 (O75594, UniProt Protein) with its neighbor proteins in the PPI network.

Protein A	Body fluid type number^a^	Protein B	Body fluid type number^a^	Interaction confidence
O75594	6, 7, 11	P61626	1, 2, 3, 4, 6, 7, 8, 10, 11	0.532
O75594	6, 7, 11	O15263	7	0.501
O75594	6, 7, 11	P05231	6	0.300
O75594	6, 7, 11	P13500	6	0.291
O75594	6, 7, 11	P60022	6, 11, 7	0.291
O75594	6, 7, 11	P01350	6	0.286
O75594	6, 7, 11	P78380	11	0.279
O75594	6, 7, 11	P07492	8	0.257
O75594	6, 7, 11	P02743	3, 6, 7, 8, 9, 10, 11	0.249
O75594	6, 7, 11	P05120	6, 7, 10	0.243
O75594	6, 7, 11	P35858	1, 3, 6, 9, 11	0.235
O75594	6, 7, 11	P49913	1, 6, 7, 8, 11	0.232
O75594	6, 7, 11	P01375	6	0.227
O75594	6, 7, 11	Q13410	4, 5	0.221
O75594	6, 7, 11	P48023	6	0.218
O75594	6, 7, 11	P19883	6	0.207
O75594	6, 7, 11	P05814	3, 4, 5	0.196
O75594	6, 7, 11	P11226	6	0.191
O75594	6, 7, 11	Q14116	6	0.162
O75594	6, 7, 11	P13236	6	0.156
O75594	6, 7, 11	P02788	1, 3, 5, 6, 7, 8, 10, 11	0.154
O75594	6, 7, 11	P13501	6	0.154
O75594	6, 7, 11	P13591	3, 6, 11	0.154

a See **Table 1** for the definition of the body fluid type number.

### Further demonstration

Now, let us demonstrate the prediction method on an independent testing dataset that contains 57 blood-secreted proteins (**Table S1**). The 11 order prediction accuracies for the 57 blood-secreted proteins by the network-based method are listed in **Table 3**. The 1^st^ prediction accuracy is 96.49%, i.e., 55 of 57 proteins were predicted to be secreted into plasma/serum in the 1^st^ prediction. And the 2^nd^ prediction accuracy is 3.51%, and all the other accuracies are 0. In other words, the first 2 predictions cover the secreted locations of all the 57 blood-secreted proteins. Apparently, the results indicate a good performance of the network-based method for secreted proteins in blood. Except the proteins in the training dataset and the 57 blood-secreted proteins, few secreted proteins in other body fluids have been found in the present researches. Therefore our method was evaluated on the blood-secreted proteins.

**Table 3:** The prediction accuracies with 11 different orders for the 57 blood-secreted proteins by the network-based method, with order 1 corresponding to the most likely prediction and order 11 the least likely prediction.

Order	Accuracy (%)
1	96.49
2	3.51
3	0
4	0
5	0
6	0
7	0
8	0
9	0
10	0
11	0

### Disease biomarker discovery

The 61 possible marker proteins listed in **Table S2** were also used to demonstrate our method. The 11 order prediction accuracies for the 61 marker proteins are listed in **Table 4**. The 1^st^ prediction accuracy is 95.08%, indicating 58 of 61 proteins were predicted to be secreted into plasma/serum in the 1^st^ prediction. The remaining 3 proteins were arranged into the plasma/serum in the 2^nd^ and 3^rd^ prediction. The collected 61 biomarkers were well arranged into the correct body fluid (plasma/serum).

**Table 4:** The prediction accuracies with 11 different orders for the 61 marker proteins by the network-based method, with order 1 corresponding to the most likely prediction and order 11 the least likely prediction.

Order	Accuracy (%)
1	95.08
2	3.28
3	1.64
4	0
5	0
6	0
7	0
8	0
9	0
10	0
11	0

Based on the quite promising results obtained through this study, we can now propos a way to discover disease biomarker in body fluids. After screening the proteins showing abnormal expression levels in various diseases and identifying their subcellular locations [11], [12], [13], [14], [15], [18], [19], they can be arranged into body fluids using our method. Therefore, suitable biomarkers, such as proteins in plasma/serum or urine can be discovered.

### Application and improvement

As is discussed above, the predicted body fluids of the first 4 orders can be regarded as the candidate locations of the secreted proteins. Biologists can focus on these body fluid candidates, which can save a lot of time and labor so as to accelerate the research progress. The predicted body fluids with the last 5 or 6 orders might be excluded for consideration owing to their low accuracies.

Considering the effectiveness of the network-based method for human secreted protein, it is possible to apply the current method to predict the locations of secreted proteins in other species. The PPI network can be collected from numerous sources including STRING [27] (Version 8.0 covered 630 organisms), worm PPI database [15], fly database [55], human PPI database [56], [57], [58], BIND [59], BioGRID [60], CYGD [61], DIP [62], HPRD [63], MINT [64], IntAct [65], and so forth. Based on the approach proposed in this paper, we can predict the body fluids for proteins of other organisms as well.

The performance of the network-based method can be further improved via the following two avenues. The first one is to collect the PPI data of high quality to exclude the false positive interaction, which was expected to improve the prediction accuracies. The second way is to collect as much PPI data as possible for constructing the PPI network, which was expected to make the method cover as many secreted proteins as possible.

### Conclusion

In this study, a multi-target model was developed for assigning the human secreted proteins to the body fluid categories based on the PPI network. Since it is the first computational method to annotate the body fluids where human protein can be secreted into, it is anticipated that the method will benefit the relevant experimental researches and stimulate a series of follow-up investigations into this emerging and challenging area.

## Supporting Information

Table S1 The 57 blood-secreted proteins used to test the network-based method.
(DOC)

Table S2 The 61 abnormally expressed proteins (possible biomarkers) used to test the network-based method that were involved with various cancers.
(DOC)
