# Supplementary material for: Prediction of Body Fluids where Proteins are Secreted into Based on Protein Interaction Network
Source: PLoS One. 2011 Jul 29;6(7):e22989. doi: 10.1371/journal.pone.0022989 (PMC3146524; doi:10.1371/journal.pone.0022989)
Supplement: Table S1 — The 57 blood-secreted proteins used to test the network-based method. (DOC) [file pone.0022989.s001.doc]

**Table S1.** The 57 blood-secreted proteins used to test the network-based method.

| Gene Name | Protein UniProt AC | Gene Name | Protein UniProt AC |
| --- | --- | --- | --- |
| PDZD2 | O15018 | EFEMP1 | Q12805 |
| SEMA3E | O15041 | PLA2R1 | Q13018 |
| ADAMTS3 | O15072 | LRP8 | Q14114 |
| ANGPTL7 | O43827 | COL19A1 | Q14993 |
| DBC1 | O60477 | PON3 | Q15166 |
| SLIT3 | O75094 | GNPTAB | Q3T906 |
| SERPINI2 | O75830 | MMEL1 | Q495T6 |
| BMP10 | O95393 | ADAMTSL2 | Q86TH1 |
| ADAMTS2 | O95450 | SERPINA11 | Q86U17 |
| COL1A1 | P02452 | SERPINA9 | Q86WD7 |
| COL3A1 | P02461 | ANGPTL5 | Q86XS5 |
| COL5A2 | P05997 | IL28RA | Q8IU57 |
| P4HB | P07237 | ITPRIP | Q8IWB1 |
| COL1A2 | P08123 | BPIL1 | Q8N4F0 |
| COL6A3 | P12111 | LILRA3 | Q8N6C8 |
| COL11A2 | P13942 | HSD17B11 | Q8NBQ5 |
| HSP90B1 | P14625 | OLFM3 | Q96PB7 |
| AMY2B | P19961 | FUCA2 | Q9BTY2 |
| COL9A1 | P20849 | PAPPA2 | Q9BXP8 |
| COL8A1 | P27658 | COL25A1 | Q9BXS0 |
| SERPINB3 | P29508 | IFIH1 | Q9BYX4 |
| COL18A1 | P39060 | MXRA5 | Q9NR99 |
| LGALS7 | P47929 | SEC63 | Q9UGP8 |
| SERPINB4 | P48594 | GNPTG | Q9UJJ9 |
| LUM | P51884 | KLK13 | Q9UKR3 |
| CRISP3 | P54108 | NENF | Q9UMX5 |
| INHBC | P55103 | ANGPTL3 | Q9Y5C1 |
| ADAMTS12 | P58397 | FCGBP | Q9Y6R7 |
| COL10A1 | Q03692 |  |  |
