# Supplementary material for: Prediction of Body Fluids where Proteins are Secreted into Based on Protein Interaction Network
Source: PLoS One. 2011 Jul 29;6(7):e22989. doi: 10.1371/journal.pone.0022989 (PMC3146524; doi:10.1371/journal.pone.0022989)
Supplement: Table S2 — The 61 abnormally expressed proteins (possible biomarkers) used to test the network-based method that were involved with various cancers. (DOC) [file pone.0022989.s002.doc]

**Table S2.** 61 abnormally expressed proteins (possible biomarkers) involved various cancers used to test the network-based method.

| Gene Name | Protein UniProt AC | Cancer Type |
| --- | --- | --- |
| SDCBP | O00560 | Melanoma [1] |
| PSCA | O43653 | Prostate cancer [2] |
| CHP2 | O43745 | HCC [3] |
| CLSTN1 | O94985 | Melanoma [1] |
| C1R | P00736 | Pancreatic cancer [4] |
| PLAT | P00750 | Renal cancer [5] |
| CA1 | P00915 | Renal cancer [5] |
| CGB | P01233 | Testicular cancer |
| TNF | P01375 | Prostate cancer [6] |
| IFNG | P01579 | Colorectal cancer [7] |
| MB | P02144 | Uterine cervix cancer [8] |
| MBP | P02686 | Brain cancer [9] |
| CSF2 | P04141 | Pancreatic cancer [10] |
| SOD2 | P04179 | Melanoma [1] |
| GAPDH | P04406 | Uterine cervix cancer [11] |
| ERBB2 | P04626 | Bladder cancer [12] |
| IL4 | P05112 | Pancreatic cancer [13] |
| IL5 | P05113 | Cervical Cancer [14] |
| F13B | P05160 | Pancreatic cancer [4] |
| HPN | P05981 | Prostate cancer [2] |
| CEACAM5 | P06731 | Gastric cancer [15] |
| KLK3 | P07288 | Prostate cancer [2], bladder cancer [12] |
| CTSD | P07339 | Breast cancer [16], Melanoma [1] |
| PFN1 | P07737 | Melanoma [1] |
| CTSB | P07858 | Melanoma [1] |
| THBS1 | P07996 | Melanoma [1] |
| ANXA5 | P08758 | Bladder cancer [12]; Melanoma [1] |
| C1S | P09871 | HCC [17] |
| IL8 | P10145 | Brest cancer [18] |
| CCL3 | P10147 | ovarian cancer [19] |
| CTSA | P10619 | Melanoma [1] |
| CHGA | P10645 | Prostate cancer [2] |
| IGF2R | P11717 | Melanoma [1] |
| CD79A | P11912 | Prostate cancer [17] |
| CCL5 | P13501 | Gastric cancer [20] |
| KRT10 | P13645 | Pancreatic cancer [4] |
| MIF | P14174 | Melanoma [1] |
| TIMP2 | P16035 | Ovarian cancer [21] |
| GLB1 | P16278 | Uterine cervix cancer [8] |
| TYMP | P19971 | Renal cancer [5] |
| KLK2 | P20151 | Prostate cancer [2] |
| TNC | P24821 | Melanoma [1] |
| IL12A | P29459 | Colon cancer [22] |
| CEACAM8 | P31997 | Lung cancer [23] |
| PMEL | P40967 | Melanoma [1] |
| MCAM | P43121 | Melanoma [1] |
| CSN1S1 | P47710 | Renal cancer [5] |
| LAMB2 | P55268 | Melanoma [1] |
| IL2 | P60568 | Kidney cancer, melanoma [34] |
| YWHAE | P62258 | Melanoma [1] |
| RAP1A | P62834 | Melanoma [1] |
| TNFAIP6 | P98066 | Lung cancer [16] |
| GOLGA3 | Q08378 | Ovarian cancer [14] |
| MFGE8 | Q08431 | Melanoma [1] |
| MVP | Q14764 | Renal cancer [5] |
| APOA1BP | Q8NCW5 | Pancreatic cancer [4] |
| C19orf10 | Q969H8 | Melanoma [1] |
| LGMN | Q99538 | Melanoma [1]; lung [24] |
| GDF15 | Q99988 | Melanoma [1] |
| DIDO1 | Q9BTC0 | Ovarian cancer [14] |
| CTSZ | Q9UBR2 | Melanoma [1] |

1. Pardo M, Garcia A, Antrobus R, Blanco MJ, Dwek RA, et al. (2007) Biomarker discovery from uveal melanoma secretomes: identification of gp100 and cathepsin D in patient serum. J Proteome Res 6: 2802-2811.

2. Bradford TJ, Tomlins SA, Wang X, Chinnaiyan AM (2006) Molecular markers of prostate cancer. Urol Oncol 24: 538-551.

3. Wang Y, Han KJ, Pang XW, Vaughan HA, Qu W, et al. (2002) Large scale identification of human hepatocellular carcinoma-associated antigens by autoantibodies. J Immunol 169: 1102-1109.

4. Bloomston M, Zhou JX, Rosemurgy AS, Frankel W, Muro-Cacho CA, et al. (2006) Fibrinogen gamma overexpression in pancreatic cancer identified by large-scale proteomic analysis of serum samples. Cancer Res 66: 2592-2599.

5. Unwin RD, Harnden P, Pappin D, Rahman D, Whelan P, et al. (2003) Serological and proteomic evaluation of antibody responses in the identification of tumor antigens in renal cell carcinoma. Proteomics 3: 45-55.

6. Goodsell DS (2006) The molecular perspective: tumor necrosis factor. Oncologist 11: 83-84.

7. Turner PK, Houghton JA, Petak I, Tillman DM, Douglas L, et al. (2004) Interferon-gamma pharmacokinetics and pharmacodynamics in patients with colorectal cancer. Cancer Chemother Pharmacol 53: 253-260.

8. Berlin Grace VM, Niranjali Devaraj S, Radhakrishnan Pillai M, Devaraj H (2006) HPV-induced carcinogenesis of the uterine cervix is associated with reduced serum ATRA level. Gynecol Oncol 103: 113-119.

9. Lamers KJ, Vos P, Verbeek MM, Rosmalen F, van Geel WJ, et al. (2003) Protein S-100B, neuron-specific enolase (NSE), myelin basic protein (MBP) and glial fibrillary acidic protein (GFAP) in cerebrospinal fluid (CSF) and blood of neurological patients. Brain Res Bull 61: 261-264.

10. Laheru D, Lutz E, Burke J, Biedrzycki B, Solt S, et al. (2008) Allogeneic granulocyte macrophage colony-stimulating factor-secreting tumor immunotherapy alone or in sequence with cyclophosphamide for metastatic pancreatic cancer: a pilot study of safety, feasibility, and immune activation. Clin Cancer Res 14: 1455-1463.

11. Lin YW, Lai HC, Lin CY, Chiou JY, Shui HA, et al. (2006) Plasma proteomic profiling for detecting and differentiating in situ and invasive carcinomas of the uterine cervix. Int J Gynecol Cancer 16: 1216-1224.

12. Sheng KH, Yao YC, Chuang SS, Wu H, Wu TF (2006) Search for the tumor-related proteins of transition cell carcinoma in Taiwan by proteomic analysis. Proteomics 6: 1058-1065.

13. Prokopchuk O, Liu Y, Henne-Bruns D, Kornmann M (2005) Interleukin-4 enhances proliferation of human pancreatic cancer cells: evidence for autocrine and paracrine actions. Br J Cancer 92: 921-928.

14. Yamazaki H, Inoue T, Tanaka E, Isohashi F, Koizumi M, et al. (2005) Pelvic irradiation-induced eosinophilia is correlated to prognosis of cervical cancer patients and transient elevation of serum interleukin 5 level. Radiat Med 23: 317-321.

15. Liu W, Liu B, Xin L, Zhang Y, Chen X, et al. (2007) Down-regulated expression of complement factor I: a potential suppressive protein for gastric cancer identified by serum proteome analysis. Clin Chim Acta 377: 119-126.

16. Dabrosin C, Johansson AC, Ollinger K (2004) Decreased secretion of Cathepsin D in breast cancer in vivo by tamoxifen: mediated by the mannose-6-phosphate/IGF-II receptor? Breast Cancer Res Treat 85: 229-238.

17. Drake RR, Schwegler EE, Malik G, Diaz J, Block T, et al. (2006) Lectin capture strategies combined with mass spectrometry for the discovery of serum glycoprotein biomarkers. Mol Cell Proteomics 5: 1957-1967.

18. Benoy IH, Salgado R, Van Dam P, Geboers K, Van Marck E, et al. (2004) Increased serum interleukin-8 in patients with early and metastatic breast cancer correlates with early dissemination and survival. Clin Cancer Res 10: 7157-7162.

19. Negus RP, Stamp GW, Hadley J, Balkwill FR (1997) Quantitative assessment of the leukocyte infiltrate in ovarian cancer and its relationship to the expression of C-C chemokines. Am J Pathol 150: 1723-1734.

20. Leung SY, Yuen ST, Chu KM, Mathy JA, Li R, et al. (2004) Expression profiling identifies chemokine (C-C motif) ligand 18 as an independent prognostic indicator in gastric cancer. Gastroenterology 127: 457-469.

21. Philip R, Murthy S, Krakover J, Sinnathamby G, Zerfass J, et al. (2007) Shared immunoproteome for ovarian cancer diagnostics and immunotherapy: potential theranostic approach to cancer. J Proteome Res 6: 2509-2517.

22. Adris S, Chuluyan E, Bravo A, Berenstein M, Klein S, et al. (2000) Mice vaccination with interleukin 12-transduced colon cancer cells potentiates rejection of syngeneic non-organ-related tumor cells. Cancer Res 60: 6696-6703.

23. Cruz PV, Wakai T, Shirai Y, Yokoyama N, Hatakeyama K (2005) Loss of carcinoembryonic antigen-related cell adhesion molecule 1 expression is an adverse prognostic factor in hepatocellular carcinoma. Cancer 104: 354-360.
